# Phytomediated selenium nanoparticles and light regimes elicited *in vitro* callus cultures for biomass accumulation and secondary metabolite production in *Caralluma tuberculata*

**DOI:** 10.3389/fpls.2023.1253193

**Published:** 2023-09-22

**Authors:** Amir Ali, Zia-ur-Rehman Mashwani, Naveed Iqbal Raja, Sher Mohammad, Juan Pedro Luna-Arias, Ajaz Ahmad, Prashant Kaushik

**Affiliations:** ^1^ Department of Botany, Pir Mehr Ali Shah Arid (PMAS) Agriculture University Arid Agriculture University Rawalpindi, Rawalpindi, Pakistan; ^2^ Biotechnology Laboratory, Agricultural Research Institute (ARI) Tarnab, Peshawar, Pakistan; ^3^ Department of Cell Biology, and Nanoscience and Nanotechnology Ph.D. Program, Center for Research and Advanced Studies of the National Polytechnic Institute, Mexico, Mexico; ^4^ Department of Clinical Pharmacy, College of Pharmacy, King Saud University, Riyadh, Saudi Arabia; ^5^ Instituto de Conservación y Mejora de la Agrodiversidad Valenciana, Universitat Politècnica de València, Valencia, Spain

**Keywords:** *Caralluma tuberculata*, callus, elicitation, selenium, nanoparticles, secondarymetabolites

## Abstract

**Introduction:**

*Caralluma tuberculata* holds significant importance as a medicinal plant due to its abundance of bioactive metabolites, which offer a wide range of therapeutic potentials. However, the sustainable production of this plant is challenged by overexploitation, changes in natural conditions, slow growth rate, and inadequate biosynthesis of bioactive compounds in wild populations. Therefore, the current study was conducted to establish an *in vitro* based elicitation strategy (nano elicitors and light regimes) for the enhancement of biomass and production of secondary metabolites.

**Methods:**

Garlic clove extract was employed as a stabilizing, reducing, or capping agent in the green formulation of Selenium nanoparticles (SeNPs) and various physicochemical characterization analyses such as UV visible spectroscopy, scanning electron microscopy (SEM), energy dispersive X-Ray (EDX) Spectroscopy, fourier transform infrared (FTIR) spectroscopy and X-ray diffraction (XRD) were performed. Furthermore, the effects of phytosynthesized SeNPs at various concentrations (0, 50, 100, 200, and 400 µg/L on callus proliferation and biosynthesis of medicinal metabolites under different light regimes were investigated.

**Results and discussion:**

Cultures grown on Murashige and Skoog (MS) media containing SeNPs (100 µg/L), in a dark environment for two weeks, and then transferred into normal light, accumulated maximum fresh weight (4,750 mg/L FW), phenolic contents (TPC: 3.91 mg/g DW), flavonoid content (TFC: 2.04 mg/g DW) and 2,2-Diphenyl-1-picrylhydrazyl (DPPH) antioxidant activity (85%). Maximum superoxide dismutase (SOD: 4.36 U/mg) and peroxide dismutase activity (POD: 3.85 U/mg) were determined in those cultures exposed to SeNPs (100 µg/L) under complete dark conditions. While the callus cultures proliferate on media augmented with SeNPs (200 µg/L) and kept under dark conditions for two weeks and then shifted to normal light conditions exhibited the highest catalase (CAT: 3.25 U/mg) and ascorbate peroxidase (APx: 1.93 U/mg) activities. Furthermore, LC-ESI-MS/MS analysis confirmed the effects of SeNPs and light conditions that elicited the antidiabetic metabolites (cumarins, gallic acid, caffeic acid, ferulic acid, catechin, querctin and rutin). This protocol can be scaled up for the industrial production of plant biomass and pharmacologically potent metabolites using *in vitro* callus cultures of *C. tuberculata*.

## Graphical Abstract

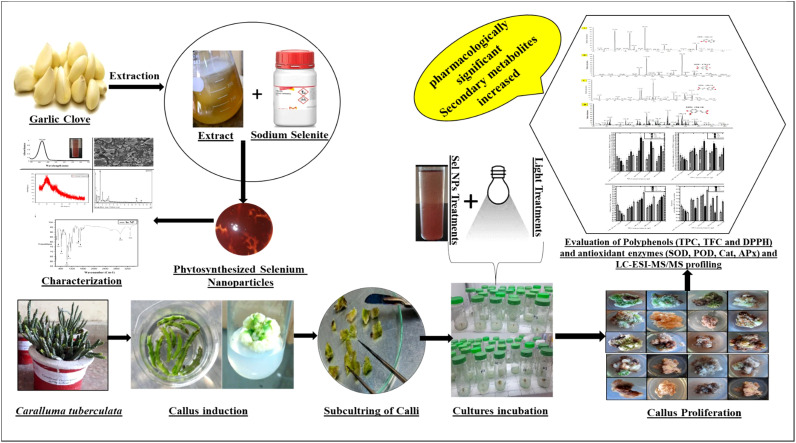

## Introduction

Plants are recognized as valuable sources of pharmaceutical compounds with various health benefits. Pharmaceutical industries are now focusing on specific compounds that play crucial roles in metabolism (Khan et al., 2019). *Caralluma tuberculata*, belonging to the family Asclepiadaceae and found in Asia, southeast Europe, Egypt, and Africa, particularly in the dry regions of Pakistan, is a remarkable pharmaceutical species. Traditionally, it has been used as a vegetable with potential antidiabetic properties due to its bitter taste. However, overexploitation from the wild for medicinal purposes can lead to the extinction of this species (Abdul-Aziz Al-Yahya et al., 2000). The pharmacological significance of *C. tuberculata* lies in its abundance of bioactive chemical compounds, such as terpenes, triterpenes, sterols, flavonoids, coumarins, gallic acid, caffeic acid, ferulic acid, and catechine (Ali et al., 2022); (Bader et al., 2003). These compounds exhibit strong antidiabetic capacity and various medicinal properties, including anti-inflammatory, antioxidant, antihyperglycemic, and antirheumatic effects. Phenolic compounds, found in specific plant organs and growth stages, play crucial roles in plant development and protection from harmful agents. Utilizing these phytomedicines could offer promising natural herbal alternatives to synthetic drugs that can harm human health (Najam-us-Saqib et al., 2013; Noreen, 2017).

Diabetes mellitus is a major health concern affecting millions of the worldwide population (Ozturk et al., 2018). A balanced diet and regular exercise are essential for managing this condition (Ogurtsova et al., 2017). Natural herbal sources rich in nutrients may help prevent oxidation caused by diabetes, presenting a safer option compared to synthetic drugs. *C. tuberculata* has gained popularity in both urban and rural populations as an effective treatment for diabetes due to its medicinal properties and nutrient-rich profile (Adnan, 2014; Ahmad et al., 2014). However, the current medicinal preparations of *C. tuberculata* are based on wild-collected plants, which can lead to variations and contamination (Alfermann and Petersen, 1995). Furthermore, less seed viability and collecting many stems for the vegetative propagation of *C. tuberculata* have become challenging. The deficient growth and biomass development of *C. tuberculata* altogether do not accomplish the marketing demands at an industrial scale. Plant tissue culture technology offers an innovative and alternative strategy for the efficient production of phytochemicals without relying on whole plants. Tissue culture allows for concentrated metabolite production, making it a preferred method in biotechnology for biosynthesizing secondary metabolites (Gaj, 2004). Researchers should focus on employing *in vitro* strategies for *C. tuberculata* to ensure a continuous supply of natural secondary metabolites.

In addition to tissue culture, nanoparticles with abiotic elicitor potential have emerged as a revolutionary strategy for synthesizing phytochemicals (Vargas-Hernandez et al., 2020). This technology provides a cost-effective and accessible means of obtaining otherwise expensive or hard-to-obtain compounds. By adopting advanced technological approaches such as tissue culture and nanoparticle-based techniques, researchers can ensure a sustainable and standardized supply of bioactive compounds, reducing the dependence on the wild collection and enhancing the potential for natural herbal remedies for diabetes and other health conditions. However, sustainable production from plants using *in vitro* culture technology to optimize growth media composition and conditions faces significant challenges (Matkowski, 2008). To address these challenges and optimize conditions for pharmaceutical vital plant production, innovative approaches leveraging the defense behavior of plants to stress can be established.

Elicitation is a widely used and cost-effective approach to induce chemical defense behavior in plants, resulting in the production of secondary metabolites. It involves the use of various physiological and molecular factors known as elicitors (Gorelick and Bernstein, 2014). By employing elicitation, the period for secondary metabolite synthesis can be reduced while maximizing productivity in low volumes of cultures (Mulabagal et al., 2004). However, the effectiveness of elicitation depends on the type, dose level, specificity of the elicitor, and cultural conditions (Vasconsuelo and Boland, 2007). Nanotechnology shows great promise in advanced biotechnology and agricultural applications (Shang et al., 2019). Nanoscale particles possess unique features such as high reactivity, wide surface area, size, and morphology, making them useful for various applications (Ramos et al., 2017). Nanoparticles (NPs) can elicit reactive oxygen species (ROS) and signaling messengers that regulate transcriptional factors related to secondary product synthesis in plants (Anjum et al., 2019). ROS serves as a significant second messenger that induces oxidative stress and activates plant metabolomics pathways (Marslin et al., 2017). However, further research is required to explore the potential usage of NPs as elicitors in plant cell cultures to promote the synthesis of secondary metabolites.

Selenium (Se) is a naturally occurring metalloid or non-metal element considered an essential micronutrient for humans and other living organisms (Zahedi et al., 2019). However, its requirement and effectiveness for many plant species need clarification. The effects of selenium on plant growth, development, biochemistry, and production depend on various factors, including selenium type, source, experimental methods, plant species, and developmental stages (Handa et al., 2019). Within an optimum dose range, selenium can accelerate growth, enhance the nutritional profile, improve photosynthetic efficiency, alter the nuclear transcription profile, activate antioxidant defense systems, regulate primary and secondary metabolism, adjust hormonal balances, and help plants adapt to adverse environmental conditions (Manjkhola et al., 2005; Babajani et al., 2019).

Light plays a crucial role in *in vitro* plant growth and the synthesis of secondary metabolites. Plant cells have an optimal light intensity range that regulates gene expression related to growth and compound production. However, different light regimes can lead to diverse responses in plant cells (Shehzad et al., 2021). NPs also exhibit exceptional catalytic action, which can be effectively harnessed through different light treatments to enhance their impact on callus and bioactive metabolites. Plant cells possess sophisticated photosensitive mechanisms that enable them to sense even subtle changes in light energy through cryptochrome and phytochrome receptors and trigger corresponding adjustments (Wang and Folta, 2013). Light also plays a crucial role in regulating the biosynthetic pathways of bioactive compounds, such as phenols, glucosinolates, and terpenoids, through the selective activation of different photoreceptors, gene modulation, transcription factors, and enzyme expression (Demotes-Mainard et al., 2016; Seiler et al., 2017). Previous studies have demonstrated the positive effect of light on plant growth and the accumulation of secondary metabolites (Wang et al., 2018; Mohammad et al., 2019; Shehzad et al., 2021; Ali and Abbasi, 2014).

Given the importance of abiotic elicitors, the main objectives of this project were to investigate the combined impact of SeNPs and various light regimes on the proliferation and production of bioactive secondary metabolites using *in vitro* callus cultures of *C. tuberculata.*

## Materials and methods

### Green synthesis of selenium nanoparticles

The green synthesis of SeNPs was performed in the Department of Botany, PMAS Arid Agriculture University Rawalpindi. For the green synthesis of SeNPs, 5 g of garlic cloves were collected and washed with tap water, followed by distilled water. The washed cloves were chopped and formed into a fine paste using a porcelain mortar and pestle. Then, the garlic clove paste was added to 400 mL of distilled water with continuous stirring and boiled covered with aluminum foil on a hot plate for 20 min. The extract was filtered through a Whatman No. 1 filter paper and kept at 4°C until its use. For the formulation of SeNPs, 20 mL of 10 mM sodium selenite (Sigma Aldrich) solution was prepared, and the garlic extract (10 mL) was added dropwise under constant magnetic stirring. Then, the solution was incubated at 36°C at 120 rpm on an orbital shaker for 4 to 6 days under dark conditions. The formation of SeNPs was demonstrated by observing a color change from uncolored to brick red. The mixture was centrifuged for 15 min at 10,000 rpm at room temperature, and the supernatant was discarded. The pellet was resuspended in 2 mL methanol and centrifuged as mentioned, and the pellet containing the purified SeNPs was dried and stored for experimentation (Anu et al., 2017).

### Physicochemical characterization of selenium nanoparticles

After visual observation, the initial confirmation of plant-based SeNPs has been confirmed through UV–visible spectrometry. The sample was prepared by immersing the SeNPs in distilled water and sonicating it for 15 min. The absorbance spectrum was noted from 200 to 700 nm through a spectrophotometer. Fourier transform infrared (FTIR) spectrometry was used to determine the functional groups in the SeNPs. Characterization was performed in the 400–4,000 cm^-1^ wave number range with an FTIR spectrometer (NICOLET 6700, Thermo, Waltham, MA, USA).

The structure characterization of SeNPs was done through scanning electron microscopy (SEM, JSM5910 JEOL, Tokyo, Japan). The SEM’s magnification was adjusted to ×10, and the scanning electron was set at 5 kV. The sample was prepared through the drop procedure, which makes use of a copper grid that has been carbon-coated. The samples were dropped onto a copper-coated grid to create a film of SeNPs. The excess solution was blotted out with blotting paper, and the film was dried for 10 min under a mercury lamp. At different magnifications, the surface topography of SeNPs was examined.

EDX detector (SIGMA model) was used to analyze the elemental composition of phytosynthesized SeNPs, which was done by using a previous protocol (Javed et al., 2020).

### Callus culture establishment

Fresh apical shoots of *C. tuberculata* were collected from potted-grown plants. The explants were cleaned with normal tap water and then sterilized with Tween20 and 1.5% sodium hypochlorite for 20 min. Furthermore, the explants were washed thrice with sterilized distilled water, followed by dipping in mercuric chloride (0.05%) for 10 min, and then cleaned with dH_2_O. All sterilized explants were transferred to growth media for callus induction using the protocol described by Ali et al. (2019), and the media composition, including MS basal salts, iron source, vitamins, 30 g/L sucrose, 7 g/L agar, and plant growth regulators (2,4D—0.5 mg/L and BA—3 mg/L), was employed. The media pH was adjusted between 5.7 and 5.8 before autoclaving for 20 min at 121°C. Finally, the cultures were placed under controlled conditions followed by 25°C ± 3°C and white light for a 16-h photoperiod. In the fourth week, the calli were used for the callus proliferation experiment.

### Collaborative impact of selenium NPs along light regimes on callus growth kinetics and proliferation

The established calli (4 weeks old) on culture media supplemented with plant growth regulators (PGRs; 2,4-D—2 mg/L and BA—2 mg/L) was subjected in the following experiments on testing the combinatorial effect of SeNPs along with different light intensities on calli biomass production. The method outlined by Ali et al. (2019) in their study was employed to create a stock solution of SeNPs. Afterward, the resultant stock solution underwent aseptic filtration using a microfilter before being introduced into the culture media. Nano-selenium at various concentrations (50, 100, 200, and 400 μg/L) was fortified with plant growth regulators. However, growth media fortified with only PGRs (2,4-D and BA, 2 mg/L each) will be employed as a control. After subculturing the calli, the cultures were shifted to the growth room and kept in the presence of various light regimes (normal light: 2,000–2,500 lx, diffused light: 500–1,000 lx, and complete darkness) for 14 days, and then the samples were finally transferred to normal light, respectively. To calculate the light intensities, a lux meter was used. To maintain completely dark conditions, the cultures were covered with black plastic sheets. The growth chamber conditions were adjusted to light for 16 h and to a dark photoperiod for 8 h, with 25 ± 1°C temperature and 70% relative humidity. The experiments were directed three times, and the progression curve represented the biomass increase under the combinatorial impact of SeNPs and light treatments. After 56 days, the total biomass data was recorded regarding callus nature, proliferation rate, and fresh weight. Data were observed every 7 days until the 56th day of the 8th week of culture.

### Evaluation of secondary metabolite profiling in *in vitro* callus cultures

#### Preparation of *in vitro*-grown cultures of *C.* t*uberculata* c*allus* extraction for phytochemical screening

To assess the elicitors’ effect on bioactive secondary metabolite accumulation, *in vitro*-proliferated callus cultures of *C. tuberculata* were employed for phytochemical examination. All developed cultures, either treated or untreated with SeNPs, were used. The proposed methodology of Khan et al. (2013) was followed, with minor amendments for extracting phytochemicals from samples. In the experiment, from each sample, approximately 300 mg powder was weighed and dissolved in 10 mL methanol (50%), shaken (24 rpm; 25°C ± 1°C) for 24 h, sonicated for 30 min, followed by vortexing for 5 min, and vigorously stirred for 15 min. Afterward, the resulting samples were centrifuged at 6,500 rpm for 10 min at room temperature. The supernatant was separated with a syringe and added to new Eppendorf tubes. The plant sample was diluted up to 10 mg/mL of final concentration for consistent analysis. The final solution was stored for further analysis at 4°C.

### Assessment of polyphenols (TPC and TFC) and total antioxidant capacity (DPPH) in callus cultures

Folin–Ciocalteu reagent was used to determine total phenolic contents (TPC) activity according to Velioglu’s protocol (Velioglu et al., 1998). In the present experiment, a total of 20 μL of sample (10 mg/mL) was loaded into each well of a 96-well plate. Then, Folin–Ciocalteu (90 μL) reagent that has been diluted 10 times was poured into the sample wells. After 5 min, the mixture was blended with sodium carbonate (90 μL), resulting in 200 μL. Methanol was the negative control, whereas gallic acid was the positive control. The absorbance was measured at 630 nm after 90 min of incubation through a Biotek microplate reader (ELX 800, BIOTEK). The outcomes were given in milligrams of gallic acid equivalent per gram. The aluminum trichloride procedure assessed the total flavonoid content (Chang et al., 2002). Approximately 20 μL of the sample (10 mg/mL) from each reaction was injected into a well on the microplate. The sample absorbance was determined at 450 nm after 30 minutes using a Biotek microplate reader. The findings were mentioned as milligram quercetin equivalent per gram.

The sample extract’s potency to detoxify the free radical DPPH was determined as published (Abbasi et al., 2010). The 96-well plate was supplied with different concentrations of the samples; standard ascorbic acid was used for the positive control group and absolute methanol for the negative control using volumes of 10, 5, 2.5, and 1 μL. The wells were then filled with 190, 195, 197.5, and 199 μL of DPPH0 solution (4.8 mg/50 mL), respectively, in a known methanol concentration. The samples’ final concentrations (1,000, 750, 500, and 250 μg/mL) were adjusted. The below-mentioned formula was followed for the determination of free radical scavenging activity.


% scavenging DPPH° free radical = (1 − AE / AD) × 100

where AE is the solution absorbance when a certain quantity of sample extract is used, and AD is the absorbance of the DPPH solution without sample extract.

### Evaluation of enzymatic antioxidant activity

Enzymatic antioxidant defense activities of desired samples were analyzed using the documented protocol described by Ali et al. (2018), with minor alterations. Approximately 2 mL (50 mM) phosphate buffer, pH 7.8, 1% polyvinylpyrrolidone, and 0.1 mM EDTA solution was used rapidly to produce a homogenate of the mortar-ground plant material. The extracts were centrifuged twice at 12,000 rpm for 15 min at 4°C temperature. The activities of superoxide dismutase (SOD), peroxide dismutase activity (POD), catalase (CAT), and ascorbate peroxidase (APx) were determined using a UV–vis spectrophotometer according to the technique of Giannopolitis and Ries (1977).

### Investigation of antidiabetic compounds using LC/ESI-MS/MS analysis

The successive fractions that showed noticeably increased callus development and antioxidant potential were further investigated to identify bioactive anti-diabetic compounds through LC–MS/MS spectrometry (Thermo Electron Corporation, USA) (Qamar et al., 2020). The direct injection mode with electron spray ionization was adopted for detection purposes, with the electron spray ionization method being used. The sample flow rate (8 uL/min), temperature (280°C), and mass range (50–1,000 m/z) were all kept constant, respectively. The collision-induced energy during MS/MS mainly depends upon the nature of the parent molecular ion, which was kept between 10 to 45. By carefully adjusting the parameters and infusing analytes, each chemical was adjusted for the MS parameters to provide the best possible ionization, ion transfer, and signal of the parent and daughter fragments. For each analyte, the source parameters were the same. Manual and Xcalibur methods were used to analyze the ESI-MS/MS data that had been obtained, and ChemDraw (ChemDraw Ultra 8.0) was used to perform structural elucidation, which was then contrasted with data that had been previously published.

## Results and discussion

### Phytosynthesis and characterization of SeNPs

**Figure 1:**
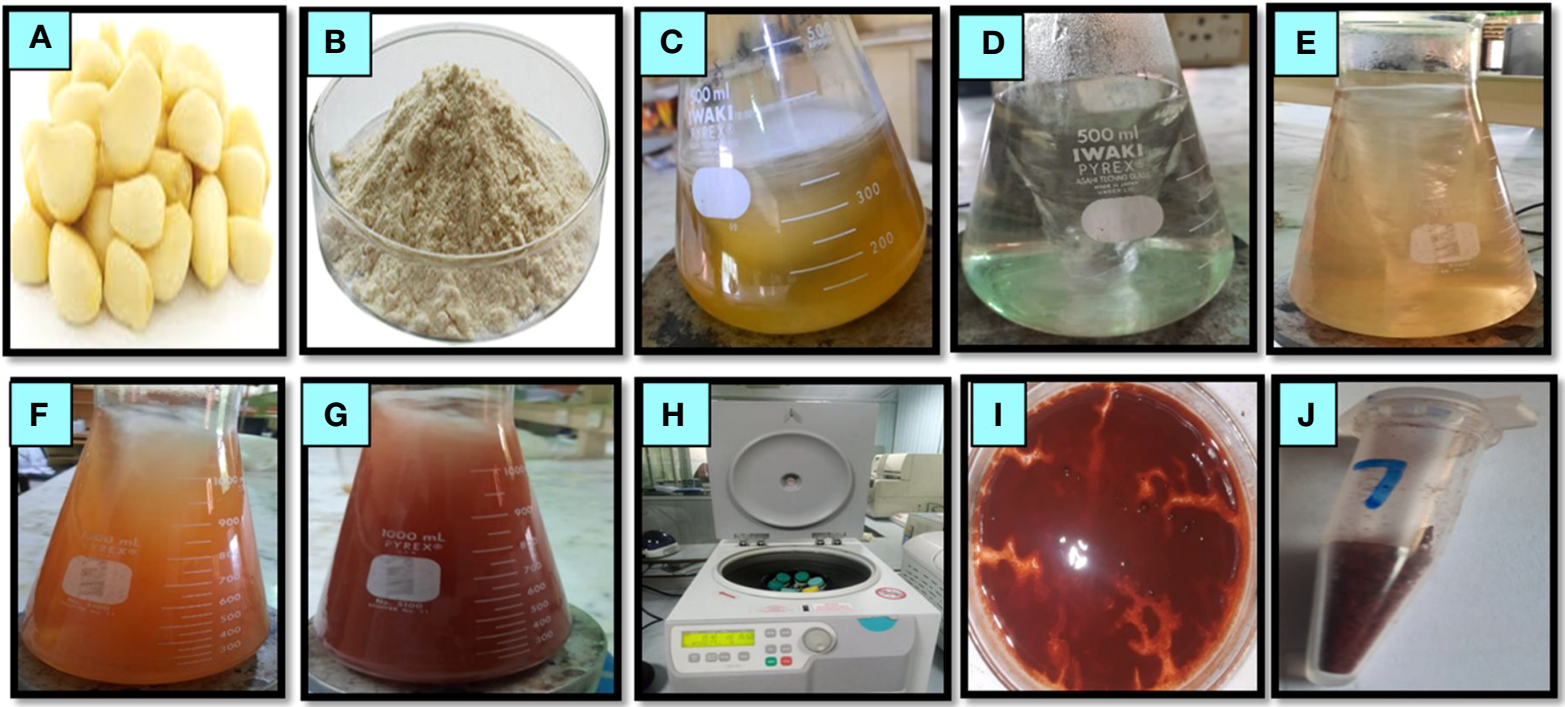
Pictorial presentation of phytosynthesized selenium nanoparticles using garlic clove extract. Garlic clove **(A)**, garlic powder **(B)**, garlic extract **(C)**, sodium selenite solution **(D)**, the addition of garlic extract to sodium selenite **(E)**, sequential color changes from light brick **(F)** to red brick **(G)**, centrifugation of the mixture solution **(H)**, collected pellet nanoparticles after centrifugation **(I)**, and selenium nanoparticles in dried powder form **(J)**.

**Figure 2:**
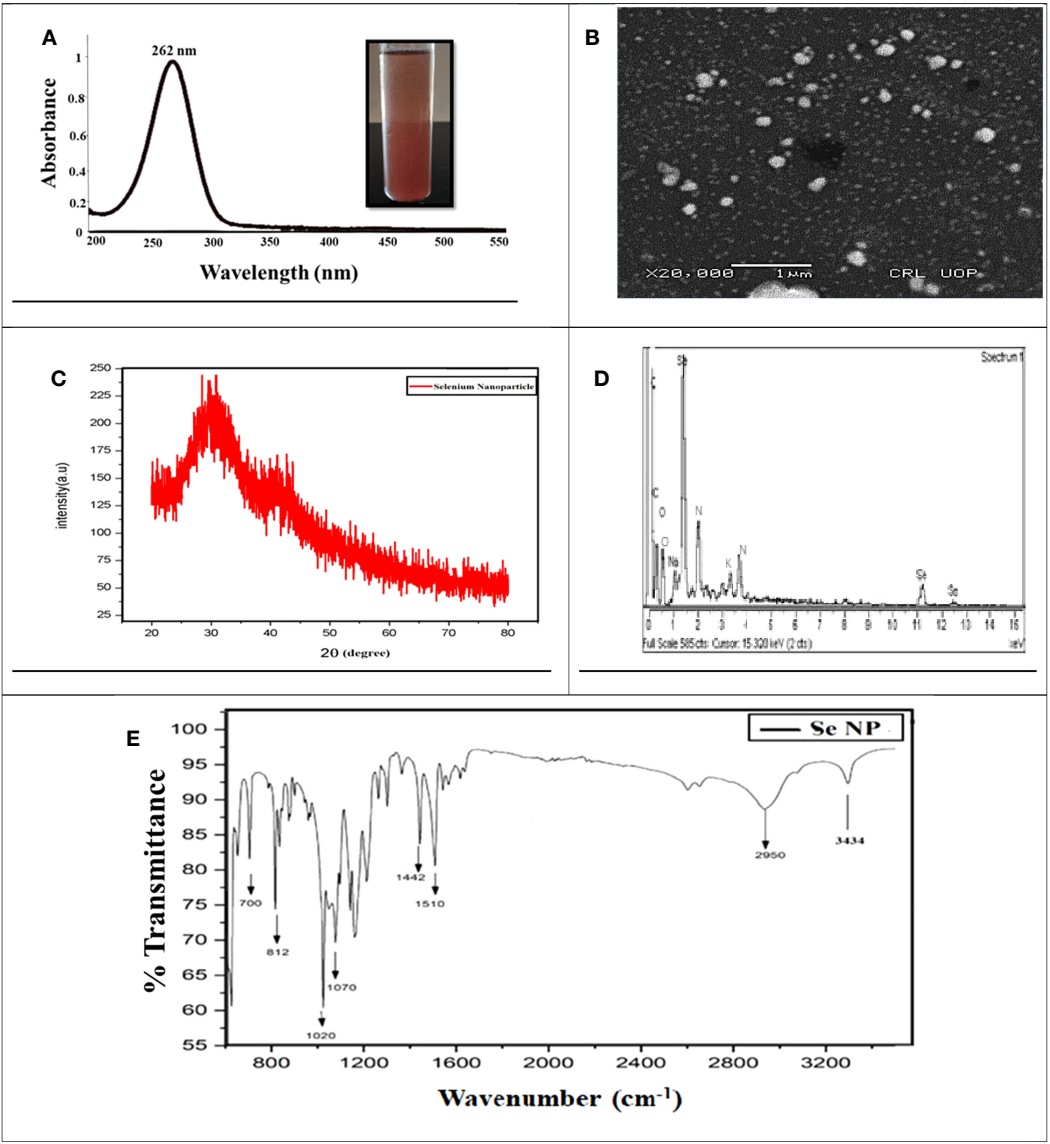
Characterization of phytosynthesized selenium nanoparticles. **(A)** UV–Visible spectrometry image of phytosynthesized SeNPs. **(B)** SEM image of phytosynthesized SeNPs. **(C)** XRD image of phytosynthesized SeNPs. **(D)** EDX image of phytosynthesized SeNPs. **(E)** The peaks at FTIR image of phytosynthesized SeNPs

Phytosynthesis of nanomaterial is more beneficial than conventional methods, such as chemical or physical methods, due to its being less toxic, eco-friendly, and cost-effective. In the current research, the results confirmed that utilizing garlic clove extract was a suitable reducing, capping, and stabilizing agent for synthesizing SeNPs. The phytosynthesized SeNP formulation was confirmed through various characterization techniques. The first confirmation of nanoparticle synthesis *via* visual observation was to change the color to brick red after adding garlic extract (**Figure 1**). Furthermore, a UV–visible spectrum (200–600 nm) was obtained to confirm the SeNP synthesis. The characterization peaks of SeNPs were recorded from the 200- to 500-nm range. The spectrum showed the peak at 262 nm, demonstrating the features of surface plasma resonance of biosynthesized selenium NPs (**Figure 2A**). A similar outcome was also acknowledged by an earlier scientific report in which garlic clove extract-mediated SeNPs displayed a characterized peak at 260 and 258 nm in the UV–visible spectrum (Anu et al., 2017; Ikram et al., 2021; Ikram et al., 2022).

To examine the reducing, capping, and stabilizing agent in the SeNP solution, FT-IR spectroscopy was conducted (Lian et al., 2019). The results demonstrated that the peaks at 3,434 cm^−1^ confirmed the existence of OH and NH groups that are playing an essential role in NP synthesis. The peak at 2,950, 1,510, and 10,220 cm^−1^ demonstrated the presence of carbon and hydrogen stretching, C=C alkene, and C-O group stretching, which could be the alcohol group certified, and specifies that clove extract makes a bond with sodium selenite. The absorption peaks at 1,442, 700, and 2,950 cm^−1^ may be recognized as the existence of aldehyde, carboxylic, oxygen, nitrogen, and amine groups. Our results suggested that garlic cloves extract enriched with functional groups were significantly involved in sodium selenite salt reduction and formed SeNPs (**Figure 2E**). In a similar study, it was also reported by Anu et al. (2017) and shown that functional groups such as C=C, O–H, N–H, and C=O are responsible for green synthesis. Moreover, a few other reports also resemble our results, concluding that N–H, C–H, and C=O functional groups are involved in forming SeNPs (Ragavan et al., 2017; Menon et al., 2021). We may predict that these functional groups play a vital role and might be considered strong stabilizing and powerful reducing agents during NP synthesis.

Moreover, the SeNPs’ size range was confirmed through scanning electron microscopy. The SEM analysis revealed the SeNPs’ size to be between 40 and 100 nm. Moreover, SEM exhibited that the shape of SeNPs was spherical (**Figure 2B**). These results relate to the previous scientific report (Dhanraj and Rajeshkumar, 2021) by evaluating the *Brassica oleracea*-mediated SeNPs and showed the same features including spherical, rectangular, and irregular shapes and a size between 10 and 25 nm. Moreover, energy-dispersive X-ray analysis (EDX) analysis revealed Selenium atomic signals along with carbon, oxygen, and sodium, confirming the presence of selenium and organic compounds in the SeNPs (**Figure 2D**).

The X-ray diffraction (XRD) spectrum of the SeNPs showed amorphous nature, and intense peaks in the XRD spectrum were noticeable (**Figure 2C**). In the XRD pattern, the presence of diffraction peaks at 20° and 30° was assigned to metallic SeNPs with a trigonal phase, respectively. Besides, some other essential peaks in the pattern might be explained by the diffraction peaks of selenium oxide, which is partly oxidized due to the existence of oxygen in the media (Yang et al., 2012; Ye et al., 2017).

### Collaborative effects of phytosynthesized selenium NPs and light regimes on callus growth dynamics and proliferation

The major goal of the present project was to scrutinize the photocatalytic role of SeNPs along with optimized PGRs under various light regimes on growth dynamics and bioactive antidiabetic metabolites in the callus culture of *C. tuberculata*. In an initial experiment, explants of *C. tuberculata* were obtained from a potted plant and cultured on MS media containing an optimized concentration of plant growth regulators (2,4-D + BA) to achieve antiseptic germplasm for a perpetual supply of explants (Ali et al., 2019). Our results noted the furthest callus induction frequency (75%) in the 28-day-old cultures. The dedifferentiation of mature plant tissue by callus induction is crucial in facilitating *in vitro* morphogenesis and achieving a feasible production of callus biomass (Tůmová et al., 2010). However, many variables, including pH, humidity, temperature, and photoperiod hours in the growth chamber and plant growth regulators’ type, level, and composition, impact *in vitro* callogenesis (Khan et al., 2020). For the callus induction, we followed our previous standard protocol regarding callus induction from a fresh apical shoot explant of *C. tuberculata* and its culture on MS media containing auxin 2,4-D, and cytokinine BA at 0.5 and 3 mg/L, respectively (Ali et al., 2019). It might be expected that the farthest growth response is due to the augmentation of optimal doses of PGRs such as auxin and cytokinin in the culture media, where the auxin hormone promotes cell division, cell elongation activity, and callus formation. In contrast, cytokinin accelerated the cell differentiation in the culture explants (Tan et al., 2021). Adil et al. (2018) also reported to have witnessed a similar case and demonstrated maximum callus induction frequency in *in vitro Cnidium officinale* cultures grown on a medium comprising 2,4-D and BA.

**Figure 3:**
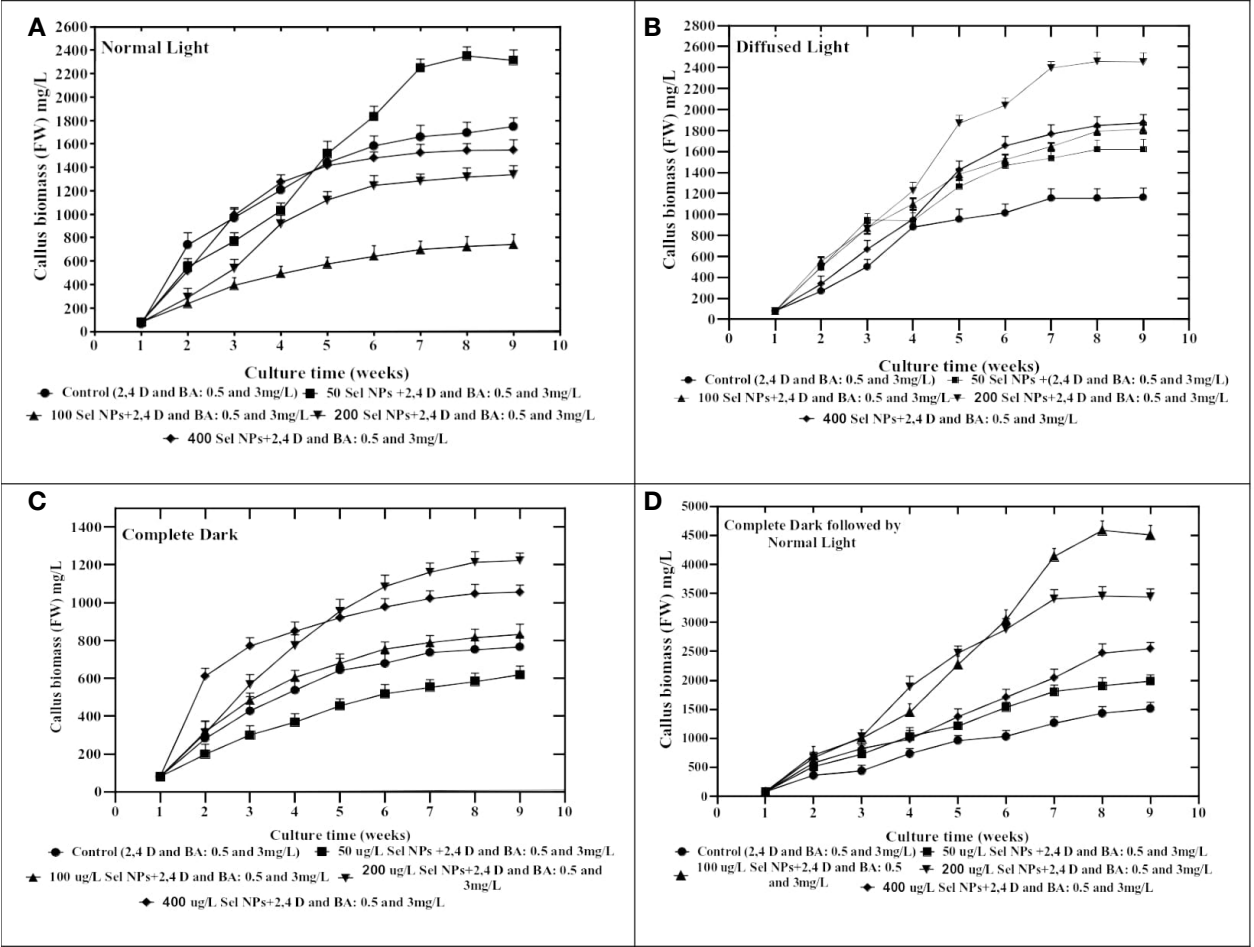
Collaborative effects of phytosynthesized selenium NPs and different light exposure doses on callus proliferation in *C. tuberculata*. **(A)** Normal Light. **(B)** Diffused Light. **(C)** Complete Dark. **(D)** Complete Dark for 2 weeks followed by normal light. Standard error ± triplicated mean values calculated for all treatments in three experiments and representing significance at *p* = .05.

After the optimization of convenient amalgamation and appropriate concentrations of 2,4-D (0.5 mg/L) and BA (3 mg/L) for callus generation, the shoot apex explant calli were exposed to MS media containing various levels of SeNPs (50, 100, 200, and 400 μg/L) along with 2,4-D (0.5. mg/mL) and BA (3 mg/L) and retained under different light intensities for callus biomass. The collaborative approach of phytosynthesized SeNPs and light regimes ensued in contradistinction growth performance in callus production and morphological features. All calli treated with different regimes were showing different responses. We perceived a considerable proliferation in callus (FW: 4,750 mg) when the callus samples were proliferated on media accompanied with 100 μg/L SeNPs + 2,4-D (0.5 mg/L) and BA (3 mg/L) and restrained for 2 weeks in a dark environment, followed by shifting to normal light (**Figure 3D**). Concerning the normal light regime, maximum growth (FW: 2,350 mg) was collected on MS media containing 50 μg/L of SeNPs + 2,4-D (0.5 mg/mL) and BA (3 mg/L), while the lowest callus biomass (1,522 mg) was collected at a higher dose of SeNPs (400 μg/L) (**Figure 3A**). In the same way, callus grown on media comprised of SeNPs (200 μg/L) and then kept under diffused light showed feasible callus growth (2,490 mg) rate. However, enhancing the dose of SeNPs (400 μg/L) along with PGRs (2,4-D and BA at 0.5 and 3 mg/L, respectively) in the media and exposure to the same condition drastically reduced the callus synthesis to 1,878 mg (**Figure 3B**). However, growth media containing 200 µg/L SeNPs, but with a completely dark environment, manufactured maximum callus proliferation (1,218 mg) (**Figure 3C**). From the perspective of essential morphological traits comprising color and texture, calli grown on media without adding SeNPs and incubated in disparate light intensities displayed a greenish and fragile nature. However, calli treated with SeNPs and retained under all selected light treatments revealed a light- to dark-brownish color with a compact nature (**Table 1**). So far, in the literature, no report is accessible on the combinatorial role of SeNPs along with light regimes in *in vitro* plant cultures to get callus proliferation, and this is the inventive exceptional report in this area of research. However, only one report is available regarding the collaborative impact of zinc oxide NPs and light sources on callus rate proliferation in *Silybum marianum*. Related to our outcomes, the highest callus biomass was attained when calli were cultured on media augmented with zinc oxide nanoparticles at 0.05 mg/L and kept for 14 days in a completely dark environment and then moved to normal light (Shehzad et al., 2021). Moreover, regarding selenium NPs, a single scientific report that justified the solitary impact of SeNPs on callus formation is available (Rajaee et al., 2020). They established an experiment to report the bitter melon seedlings’ response toward nano-selenium elicitor (0, 1, 4, 10, 30, and 50 mgL^-1^) in *in vitro* conditions and observed that a low concentration of nano-selenium enhanced the biomass accumulation, but when the concentrations were 10 mgL^-1^ and above, these caused severe toxicity. The reported data depicted that the use of nano-selenium substantially changed cell growth in a dose-dependent way, metabolomics profiling, and molecular pathways among various plant species, including *Brassica juncea* (Handa et al., 2019), *Triticum aestivum* (Safari et al., 2018), *Melissa officinalis* (Babajani et al., 2019), peppermint (Nazerieh et al., 2018), and sorghum (Djanaguiraman et al., 2018). The supplementation of phytosynthesized selenium NPs in the growth medium tremendously affects the morphological, anatomical, and antioxidant profile in an optimum applied dose manner. The consequences of the exposure of *Melissa officinalis* and *Capsicum annuum* to nano-selenium affected the plant growth productivity and metabolomics profiling (Sotoodehniakorani, 2018; Babajani et al., 2020). This can be endorsed by the differential physicochemical features of SeNPs defining their uptake, transportation, translocation, and interaction with macromolecules and cellular organelles. Furthermore, several other factors, including the synthesis method (Abbasi et al., 2020), suitable concentration, and various biological systems, showed different beneficial functions and cell toxicity against nano products (Moghanloo et al., 2019). In this concern, convincing evidence has been reported in that the uptake of nano-selenium and its consequent metabolism in different plant cells vary from the control group. Similar to our results, SeNPs at 100 µg/L were found suitable for callus growth. Alharby et al. (2016) indicated that applying ZnO NPs (30 mg/L) elicited callus formation in *Solanum lycopersicum*. An increase in the concentration of ZnO NPs from 0.1 to 100 mg/L in growth media suits a feasible amount of callus in *Stevia rebaudiana*. A lower dose of ZnO NPs at the value of 0.01 mg/L drastically reduced the callus production in *S. rebaudiana (* Javed et al., 2018).

**Table 1:** Collaborative effects of phytosynthesized selenium NPs and different light exposure doses on callus morphology in *C. tuberculata*.

Media composition	Culture incubation condition			
	Normal light: 2000–2,500 lx	Diffused light: 500–1,000 lx	Complete dark: 0 lx	Shifting of cultures from complete darkness to normal light after 2 weeks
2,4D + BA (0.5 + 3 mg/L)	Green; friable	Light yellow; friable	Yellow; friable	Light brown; friable
50 µg/L SeNPs + 2,4D + BA (0.5 + 3 mg/L)	Green; friable	Light yellow; friable	Yellow; friable	Light brown; friable
100 µg/L SeNPs + 2,4D + BA (0.5 + 3 mg/L)	Yellowish green; friable	Light brown; friable	Light brown; friable	Light brown and friable
200 µg/L SeNPs + 2,4D + BA (0.5 + 3 mg/L)	Yellowish brown; compact	Yellow; compact	Yellow; friable	Light brown; compact
400 µg/L SeNPs + 2,4D + BA (0.5 + 3 mg/L)	Brown; compact	Yellow; compact	Yellow; compact	Dark brown; compact

Applying metal NPs against environmental stresses acting as strong elicitors which upregulate the plant’s defensive mechanisms and increase the biomass is an innovative approach. In one of the latest research, silver nanomaterials were used to enhance C. tuberculata *plant biomass in* in vitro cell cultures (Ali et al., 2019). The outcomes documented that various dose levels of AgNPs and PGRs were used alone and in combination for the plant biomass production of in vitro callus cultures of C. tuberculata. The study’s findings showed that the optimum concentration of silver nanoparticles and PGRs (2,4-D and BA) in the culture media considerably improved the plant callus proliferation rate and showed a synergistic effect. Evaluating the safety and cytotoxicity of SeNPs leads to a clear comprehension of their uptake by various plants, which is relevant to the selenium NP uptake and its translocation. In this case, it is crucial to comprehend SeNP uptake and translocation. The significance of this process is still not completely understood. Several directions have been set to better understand SeNP uptake and penetration into plant biological systems (Hu et al., 2018). SeNPs penetrate the cell wall and then pass through the plasma membrane. The most frequently accepted argument for the synthesized NPs’ translocation is that these nano-sized particles can penetrate different plant cells until they enter the xylem (Behbahani et al., 2020). Once SeNPs have entered the plant’s vascular machinery, tailored nanomaterials may be delivered to the aerial parts in addition to the plant’s water transpiration and nutritional flow for the transportation of nutrients. Another additional argument for the validity of this study’s findings was that each callus formed from cultures exposed to nano-selenium differentiated differently from control groups. These changes in callogenesis could be an indirect result of the application of selenium nano elecitors, endogenous phytohormones, epigenetics, and or redox state. Assuming that during callogenesis, cell division, development, and proliferation are stimulated, the differential callogenesis seen in this investigation may be caused by alterations in endogenously produced plant hormones such as auxin, cytokinin, and abscisic acid, which are induced by nano-selenium elicitors by the NO/H_2_S signaling pathways. To clearly explain the precise aspects, further research is needed to know about how SeNPs affect cell growth at the molecular level (Rajaee et al., 2020; Sotoodehnia-Korani, 2020).

**Figure 4:**
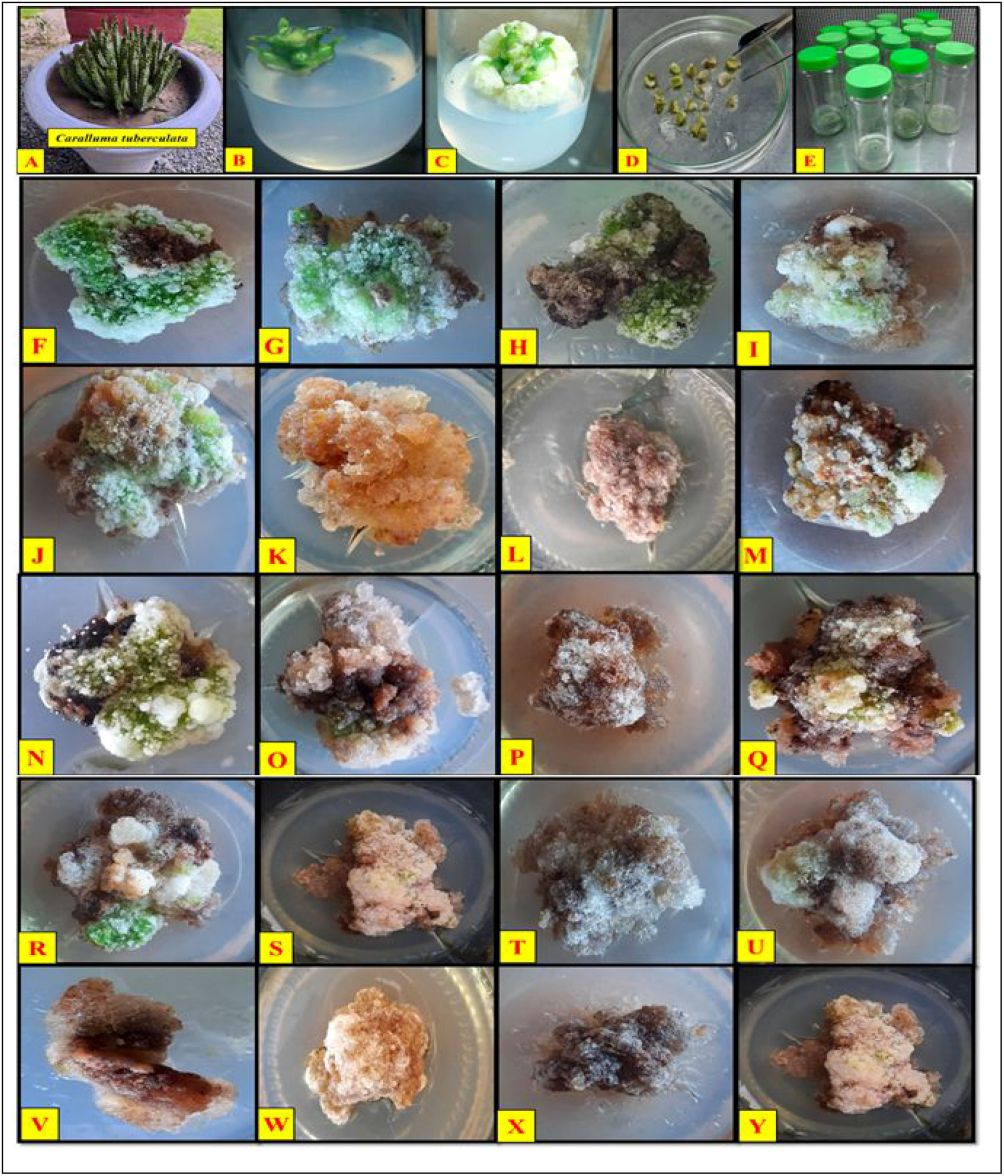
Collaborative effects of phytosynthesized selenium NPs and different light exposure doses on callus proliferation in C*aralluma* tuberculata. **(A)** *C*. *tuberculata* potted plant, **(B)** shoot apex explant culture, **(C)** callus induction, **(D)** subculturing of calli, **(E)** incubation of cultures, **(F)** 2,4-D (0.5) and BA (3 mg/L) under normal light, **(G)** 2,4-D (0.5 mg/L) and BA (3 mg/L) under diffused light, **(H)** 2,4-D (0.5 mg/L) and BA (3 mg/L) under complete darkness, **(I)** 2,4-D (0.5 mg/L) and BA (3 mg/L) under complete darkness for 2 weeks followed by normal light, **(J)** SeNPs (50 µg/L) + 2,4-D (0.5 mg/L) and BA (3 mg/L) under normal light **(K)** SeNPs (50 µg/L) 2,4-D (0.5 mg/L) and BA (3 mg/L) under diffused light, **(L)** SeNPs (50 µg/L) 2,4-D (0.5 mg/L) and BA (3 mg/L) under complete darkness, **(M)** SeNPs (50 µg/L) + 2,4-D (0.5 mg/L) and BA (3 mg/L) under complete darkness for 2 weeks followed by normal light, **(N)** SeNPs (100 µg/L) + 2,4-D (0.5 mg/L) and BA (3 mg/L) under normal light, **(O)** SeNPs (100 µg/L) + 2,4-D (0.5 mg/L) and BA (3 mg/L) under diffused light, **(P)** SeNPs (100 µg/L) + 2,4-D (0.5 mg/L) and BA (3 mg/L) under complete darkness, **(Q)** SeNPs (100 µg/L) + 2,4-D (0.5 mg/L) and BA (3 mg/L) under complete darkness for 2 weeks followed by normal light, **(R)** SeNPs (200 µg/L) + 2,4-D (0.5 mg/L) and BA (3 mg/L) under normal light, **(S)** SeNPs (200 µg/L) + 2,4-D (0.5 mg/L) and BA (3 mg/L) under diffused light, **(T)** SeNPs (200 µg/L) + 2,4-D (0.5 mg/L) and BA (3 mg/L) under complete darkness, **(U)** SeNPs (200 µg/L) + 2,4-D (0.5 mg/L) and BA (3 mg/L) under complete darkness for 2 weeks followed by normal light, **(V)** SeNPs (400 µg/L) + 2,4-D (0.5 mg/L) and BA (3 mg/L) under normal light, **(W)** SeNPs (400 µg/L) + 2,4-D (0.5 mg/L) and BA (3 mg/L) under diffused light, **(X)** SeNPs (400 µg/L) + 2,4-D (0.5 mg/L) and BA (3 mg/L) under complete darkness, **(Y)** SeNPs (400 µg/L) + 2,4-D (0.5 mg/L) and BA (3 mg/L) under complete darkness for 2 weeks followed by normal light.

The differential light source in *in vitro* plant culture plays a pivotal role by impacting the plant architecture, growth, and developmental process. In the current survey, all applied light regimes tremendously affect the growth traits of callus. When calli were subjected to the dark environment for 2 weeks and then incubated in normal light, they revealed a considerable quantity of growth in contrast with other light regimes. The most important role of pre-exposure of culture to dark conditions might influence the growth rate by regulating physiological and metabolomics profiling of plant cells. Based on the findings of Adil et al. (2019), calli were elicited by pretreatment of dark conditions relative to other light intensities. The present study outcomes align with the results of Bogale (2018), where the highest growth rate was observed under a dark environment and then exposure to normal light *in Colocasia esculenta* cultures. The currently published report also justified the importance of pretreatment of dark conditions and noticed that in initially exposed olive calli for 1 week in the dark, and then the calli samples were subjected to normal light and feasible production (Mohammad et al., 2019). On the contrary, dark treatment significantly elicited callus growth in *A. bracteosa* compared to those cultures raised under normal light conditions (Ali et al., 2018). Moreover, in the perspective of callus features, including color and texture, it was confirmed that the callus raised to culture media without supplementation of SeNPs and then all applied with light regimes turned out green-yellowish and fragile (**Table 1**; **Figure 4**). On the other hand, the interactive effect of SeNP-augmented growth media and all light treatments produced light- to dark-brownish samples with a compact nature. These differences in callus nature might be correlated to the cell division process (Adil et al., 2019). It was stated that the normal light environment in the growth chamber generated brown and soft samples, whereas the dark treatment accelerated the synthesis of yellowish and compact calli (Ali et al., 2018). It can be postulated that the brownish calli might be due to the excessive production of polyphenols due to severe stress environment by the combinatorial impact of SeNPs and light regimes. These phenolic metabolites may possibly suppress the growth and therefore trigger necrosis in the callus (Khan et al., 2019).

### Collaborative effects of phytosynthesized selenium NPs and light regimes on bioactive secondary metabolites in callus cultures

**Figure 5:**
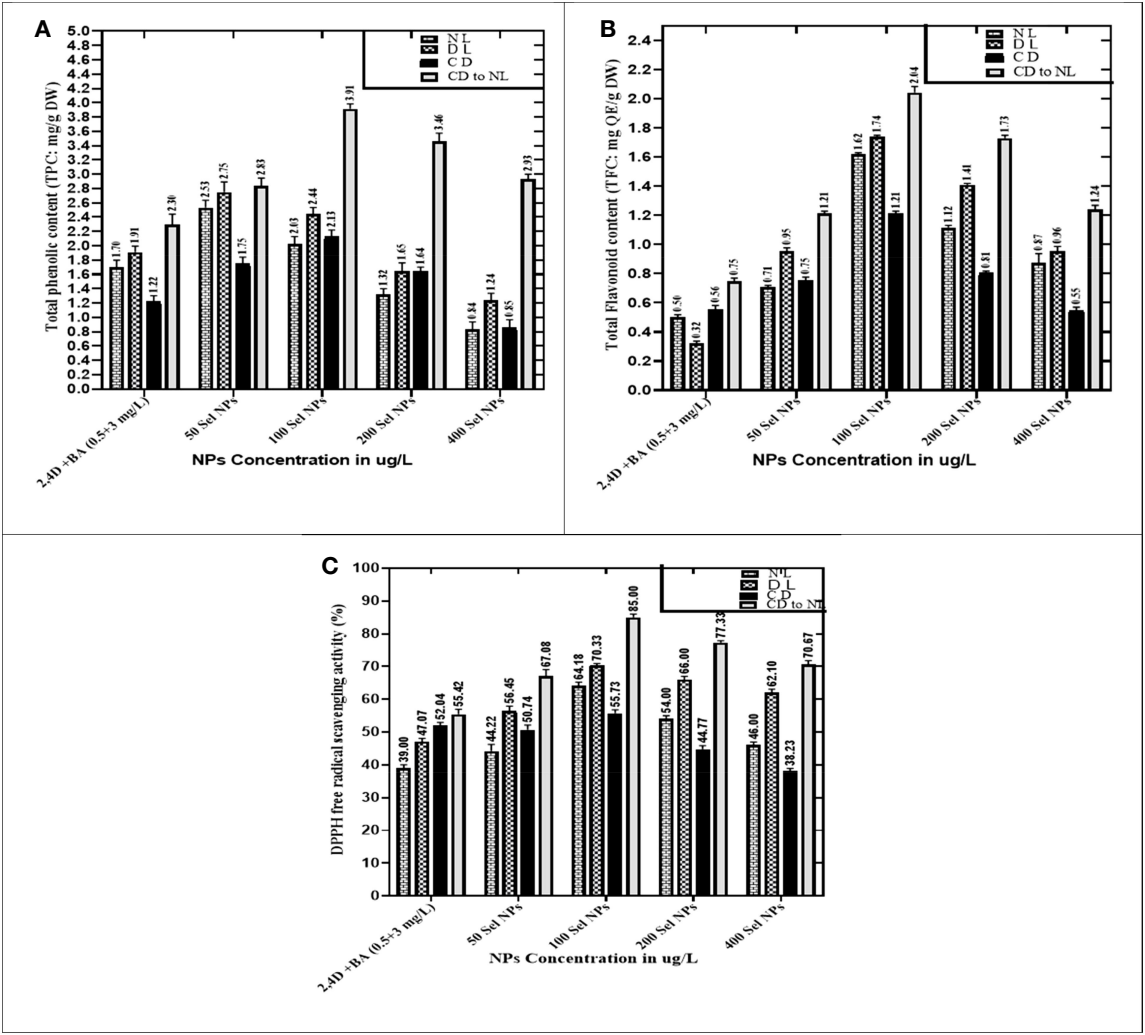
Collaborative effects of phytosynthesized selenium NPs and different light exposure doses on secondary metabolite production in callus cultures. **(A)** Total phenolic contents, **(B)** total flavonoid contents, and **(C)** DPPH free radical scavenging activity. The data represent the mean values of triplicates ± standard error. Data in columns with different letter/s differ significantly at *p* = .05.

The relationships of phytosynthesized SeNPs at various ranges with the different light regimes greatly stimulated the TPC, TFC, and DPPH antioxidant activity, respectively. The maximum TPC (3.91 mg), TFC (2.04 mg), and DPPH antioxidant activities (85%) were seen in calli that were treated with 100 μg/L SeNPs and primarily cultured for 2 weeks in a dark environment and then subjected to normal light. This was done by complete darkness, exposing these samples to light intervention, while calli raised at the highest dose of SeNPs (400 μg/L) under normal light treatment expressed the lowest activation of these polyphenols and antioxidant activities (**Figure 5**). Moreover, proliferated calli at 100 μg/L SeNPs and normal light conditions exhibited the highest TPC (2.03 mg) and TFC (1.62 mg) as compared to those cultures cultured on growth media augmented with SeNPs (400 μg/L) under the same light condition but synthesized less amount of TPC (0.84 mg) and TFC (0.87 mg). For diffused light and completely dark, maximum accumulations of TPC and TFC were noticed in the callus grown on media containing 100 μg/L SeNPs relative to other NP treatments (**Figure 5**), while increasing the dose to 400 μg/L of SeNPs in culture media expressed minimum TPC (0.85 mg) and TFC (0.55 mg) and DPPH activity (38.23%) under a dark environment, respectively.

**Figure 6:**
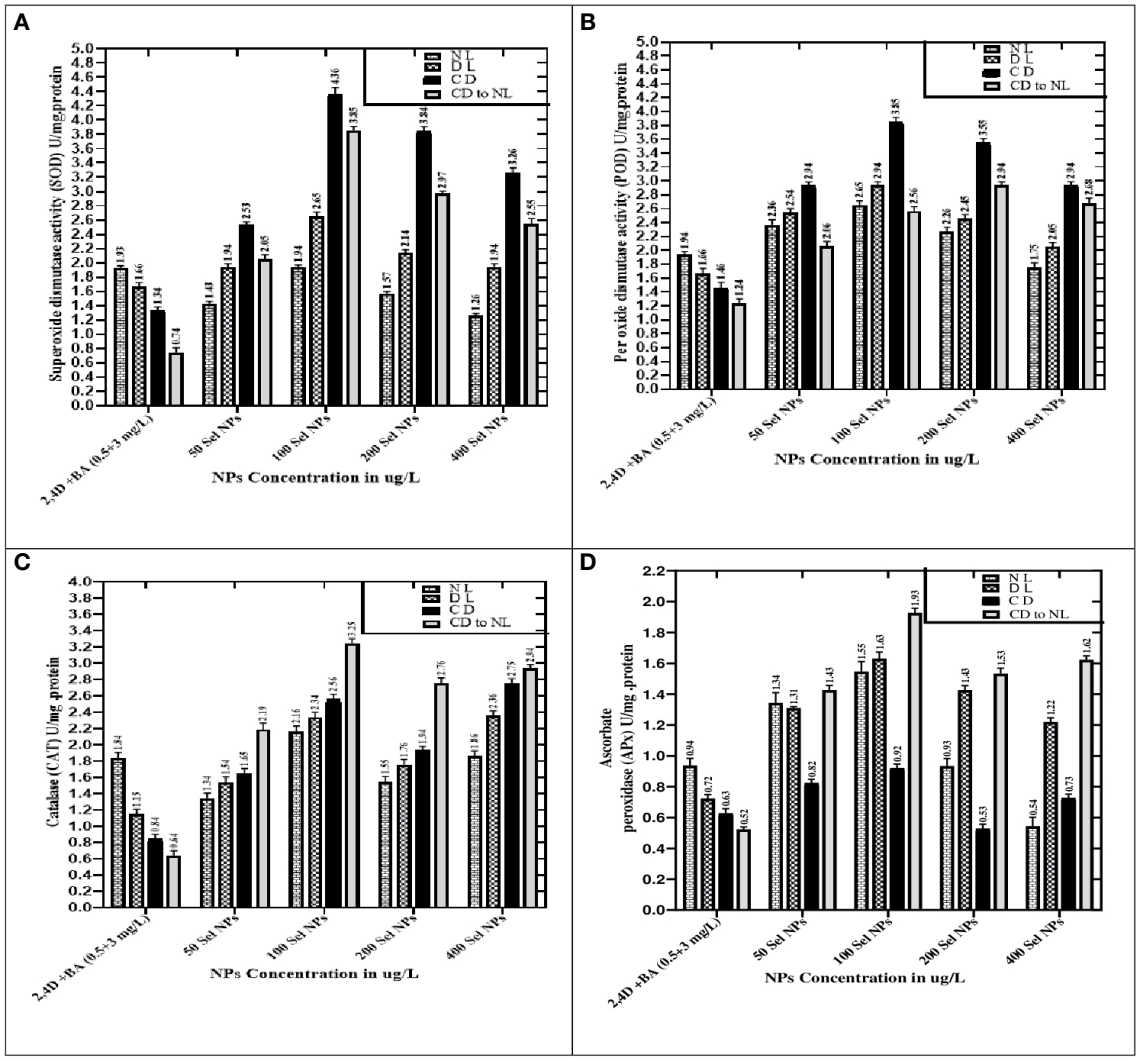
Collaborative effects of phytosynthesized selenium NPs and different light exposure doses on the production of enzymatic antioxidant activity. **(A)** Superoxide dismutase activity, **(B)** peroxidase activity, **(C)** catalase activity, and **(D)** ascorbate peroxidase activity. Data represent the mean values of triplicates ± standard error. Data in columns with different letter/s differ significantly at *p* = .05.

When the callus of different samples was cultured on media augmented with 100 μg/L SeNPs in total darkness, followed by a dark environment switching to diffused and normal light regimes, the highest production of enzymatic antioxidants like SOD (4.36 U/mg) and POD (3.85 U/mg) was observed. Furthermore, untreated calli (control treatment) showed maximum expression of SOD and POD activity (1.93 and 1.94 U/mg, respectively) under normal light intensity in the growth room, and a slight decrease was detected in diffused light and then in dark conditions (**Figure 6**).

When calli were exposed to 100 µg/L SeNPs and given a dark environment for 2 weeks and then moved to normal light conditions, we found tremendously accelerated activities of CAT of and APx (3.25 and 1.93 U/mg, respectively), followed by complete darkness, diffused light, and normal light conditions, respectively. Untreated samples were maintained in normal light conditions, and maximum antioxidant activities of CAT (1.84 U/mg) and APx (0.94 U/mg) were found, while a moderate reduction was seen in samples from a light environment followed by total darkness (**Figure 6**). Currently, one of the most acceptable strategies to achieve feasible production of phytoconstituents at an industrial scale is *in vitro* culture elicitation (Wang et al., 2004; Ali et al., 2018). However, bioactive metabolite synthesis is based on the application of elicitors in *in vitro* cultures. For that reason, optimizing suitable culture composition and a favorable environment is essential to get the considerable biomass of various bioactive secondary metabolites. To accomplish this, the fresh apical shoot-derived calli were subjected to SeNPs and different light treatments. In our experiments, the collaborative role of SeNPs at various levels and light regimes intensely expressed the highest TPC, TFC, and DPPH free radical scavenging potential of plant samples. In previous reports, *Stevia rebaudiana* calli elicited with ZnO NPs (100 mg/L) under normal light, producing the highest TPC and TFC (5.65 and 2.85 g/mg of DW) (Javed et al., 2018). In another investigation, augmenting ZnO NPs in media increased polyphenols, i.e., TPC and TFC (22.12 and 12.18 mg and 6.38 and 2.7 mg) in *in vitro Verbena tennuisecta and Verbena officinalis* cultures, correspondingly (Siddique Afridi et al., 2018). Current outcomes showed that control treatment (2,4-D and BA, 0.5 and 3 mg/L each) dropped the synthesis rate TPC and TFC under normal light. However, the optimal range of Se NPs at 100 μg/L and exposure to a dark environment for 2 weeks, then shifting to a normal light environment, fabricated extreme amounts of polyphenols.

We also noticed a similar behavior for DPPH activity, wherein SeNPs and all light treatments showed significant differences. Callus elicited with SeNPs at a low dose (100 μg/L), raised in a fully dark environment for 2 weeks, and then exposed to normal light exhibited significant DPPH activity. Javed et al. (2018) also recorded a similar effect by employing ZnO NPs at lower doses, which accelerated DPPH activity (85.91%) in *in vitro Stevia rebaudiana*. Previous scientific studies validated the photocatalytic efficiency of NPs, which showed a strong response to light and dramatically induced the collaboration of NPs with plant cells (Fouda et al., 2022). The treatment of SeNPs in the *in vitro* culture creates an oxidative environment surrounding the cell and produces reactive oxygen species. To neutralize the toxic effect, various bioactive antioxidant-defensive compounds are upregulated by plant cells for defense purposes. Owing to the strong penetrating capacity in the cell and high surface area of the small nano size, they can easily move through apoplastic and symplastic pathways, which may result in additional electrostatic interactions with cell membranes. As a result, they activate the pathways which produce polyphenols and speed up the rate of their products in the cell (Rani et al., 2017; Zhang et al., 2022).

Moreover, compared to the control group, applying SeNPs and PGRs in the culture media significantly increased the SOD and POD activity, respectively, when exposed to various light stress environments. The highest expression of SOD and POD activities was noticed in the calli raised at 100 μg/L of SeNPs under a completely dark environment. These outcomes raise interesting phenomena and confirmed the photosensitizer nature of selenium and might create electron–hole pairs in the light environment. Selenium can produce a large bandgap that cannot be able to harvest light at a significant level under high light intensity (Mellinas et al., 2019). Interestingly, this phenomenon was observed significantly in our outcomes, where SeNPs under dark conditions proved to be suitable for stimulating the SOD and POD activity compared to other light regimes. SOD is one of the essential antioxidant enzymes found in plants. It is a widely recognized metalloprotein enzyme that catalyzes the conversion of superoxide free radicals to hydrogen peroxide (Ali et al., 2018). Its stimulation may protect plant cells in stressful conditions from oxidation (Kazmi et al., 2019; Kohli et al., 2019; Kaya et al., 2020). Furthermore, light intensities and exposure timing are also considered promising factors that influence enzymatic antioxidant profiling in both *in vitro* and *in vivo* techniques (Khan et al., 2013).

The outcomes of the present project are very promising, and they revealed that there is an outstanding capability by employing SeNPs in collaboration with light regimes to accelerate the enzymatic antioxidant activities (CAT and APx) in *C. tuberculata* callus. Compared to the results of SOD and POD, opposite trends were noticed in the CAT and APx activities, and it was revealed that SeNPs at 100 μg/L in growth media in complete darkness for 2 weeks and then subjected to normal light perfect treatment get the maximum quantity of CAT and APx in callus cultures, while the outcome range of other samples followed by diffused, normal, and complete darkness regimes, respectively. However, augmentation of SeNPs at high doses along with PGRs drastically declines the CAT and APx activity in calli kept in all applied light treatments.

Effective SeNPs elicit and stimulate the gene expression of plant stress signaling pathways (Ghorbanpour and Hadian, 2015). Our results indicated that the optimum concentration of less oxidative SeNPs elicitors alters the synthesis of antioxidants, such as polyphenols, and modifies the expression of enzymes which ultimately reduce oxidative stress and help in other damaging effects of abiotic stresses. However, the difference was observed in responses depending upon the applied concentration of SeNPs. Even though SeNPs could induce the ROS in various plant cells and cause oxidative stress (Rajaee et al., 2020), because they can translocate and accumulate into the various cell organelles, this contributes to the induction of antioxidant enzymes to ameliorate oxidative pressure that may help in the improvement of plants’ performance (Hatami et al., 2017; Mansoor et al., 2022).

However, novel effective nano-elicitors’ mechanisms and ways of action for the potent production of secondary compounds vary according to their source, concentration, nutritional specificity, physiochemical conditions, and growth absorption of plants. In the elicitation mechanism, various transcription factor families regulate the interactive pathways such as mitogen-activated protein kinases phosphorylation, phenylpropanoid, reactive oxygen species burst, and calcium flux, which are early measures initiated in most of the presence of elicitor and triggers the biosynthesis of metabolite pathways (Zhao et al., 2016; Ahmad et al., 2019; Faizan et al., 2021). After the activation of transcription factors and signal transducing pathways, there is a transformation of phenylalanine into 4-coumaroyl-CoA which interacts with the plant flavonoid synthetic pathways reported (Schluttenhofer and Yuan, 2015). The receptors which are present on the surface of plasma membranes identify and then bind that specific elicitor and trigger the cascades like activation of ion fluxes NADPH oxidase, Ca^2+^ burst, ROS burst, MAPK phosphorylation, and G-protein and cytoplasmic acidification (Zhao et al., 2005). The plant immediately responds by exchanging ions, such as K^+^/Cl effluxes and Ca^2+^/H^+^ influxes. One of the most crucial events is Ca^2+^ influx, which affects the cell’s physiological functions (Maeda et al., 2005). Numerous intracellular calcium proteins, which include calmodulin-like proteins, calcium-dependent kinases, calmodulin, and phospholipases, along with secondary messengers such as diacylglycerol and inositol 1,4,5-triphosphate IP3 are affected by these Ca^2+^ signals (White and Broadley, 2003). Plant physiological functions and cellular activities such as oxidative burst regulation, hormone transduction, and gene expression are stimulated by Ca^2+^/calmodulin-mediated mechanisms. SeNPs can produce ROS after uptake and permeation into the plant cell, and their increased concentration could be detrimental to plants. Their generation may serve both in the determination of stress and protection against them. ROS-neutralizing mechanisms include protective molecule induction against stress such as polyphenols and antioxidative enzymes that alter the cells from a stressed phase to a favorable condition (Choudhary et al., 2020). In the present work, a significant improvement was observed in polyphenols and enzymatic antioxidants in the *C. tuberculata* callus culture that were augmented with SeNPs along with PGRs as compared to those cultures which were only exposed to PGRs.

### Evaluation of antidiabetic compounds in callus cultures by LC/ESI-MS/MS

Conventionally, *Caralluma* species have been broadly utilized as a traditional herbal drug to treat diabetes mellitus disease. Previous scientific investigations have discovered a wide range of potent pharmaceutical metabolites that can function as secure and efficient antidiabetic agents. Therefore, in the present research, we used LC/ESI-MS/MS analytic technique to quantify and identify antidiabetic metabolites. The antidiabetic compounds were assessed and quantified in the selected callus extract samples. The results exhibited that seven potent antidiabetic compounds (coumarins, gallic acid, caffeic acid, ferulic acid, catechine, quercetin, and rutin) were identified in the successive fraction by using the 50% methanolic fraction (**Supplementary Figure 1**). The induction of oxidative environment to *in vitro* callus cultures by using a nano elicitor (SeNPs 100 μg/L) under different light regimes considerably increased the bioactive compounds. The highest amount of antidiabetic compounds was quantified in calli treated with SeNPs at 100 μg/L and kept in total darkness for 2 weeks before being exposed to normal light (**Table 2**). These feasible fabrications of bioactive antidiabetic compounds could be endorsed to the plant defensive machinery for oxidative species scavenging and thus to survive in a stress condition. The optimistic correlation of polyphenols and identified antidiabetic compound production plays a vital function in neutralizing the generation of reactive oxygen species and provides a valuable platform for culture growth at a considerable level, while the media that lacked SeNPs and were incubated in normal conditions did not enhance the antidiabetic contents. To date, no data related to the evaluation of antidiabetic profiling of SeNPs and various light condition treatment of callus of *C. tuberculata* has been available. However, in a previous study, a similar fashion but with a different type of NPs showed that the higher dose of ZnO NPs (0.15 mg/L) increased the silymarin level in *Silybum marianum* callus under complete darkness for 14 days and which was then moved to normal light (Shehzad et al., 2021). Some of the essential bioactive compounds (gallic acid, tannic acid, coumarin, hesperidin, rutin, and quercetin) were also increased significantly in response to AgNP-treated callus of *Juniperus procera in vitro* (Salih et al., 2022). Moreover, previous reports also acknowledged the significant role of these secondary metabolites which act as antidiabetic agents by increasing the lipid profiles’ ability to absorb glucose and regulate the level of sugar in the blood, which are all important biochemical aspects of blood glucose levels (Abdel-Sattar et al., 2018; Bourhia et al., 2020). Achievements in the present work concerning the use of phytosynthesized SeNPs and light conditions in *in vitro* plant culture provide a promising platform by using new-age nanomaterials as elicitors and the possibility of creating nano environments to protect and enhance the biomass of the highly endangered *C. tuberculata* species, which may be a significant source of bioactive antidiabetic compounds. This study can serve as an excellent model for improving the production of bioactive metabolites for the pharmaceutical sectors in *in vitro* cultures of medicinal plants.

**Table 2:** Antidiabetic compounds identified in the *in vitro*-raised callus extract of *Caralluma tuberculate*.

Compound type	Molecular formula	Molecular weight	Structure	Plant material	Concentrations (μg/mg)
Coumarin	C9H6O2	146.14	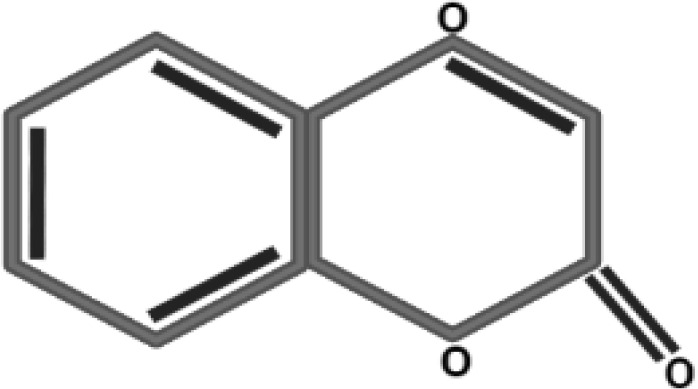	2,4D + BA (0.5 + 3 mg/L)	0.5 µg/mg
				100 µg/L SeNPs + 2,4D + BA (0.5 + 3 mg/L)	0.86 µg/mg
Gallic acid	C_7_H_6_O_5_	170.12	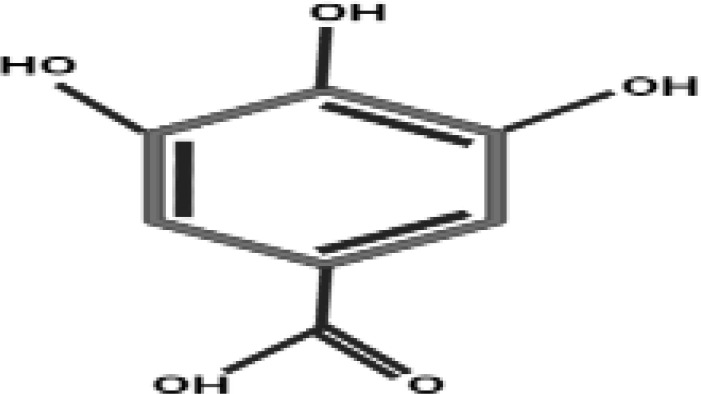	2,4D + BA (0.5 + 3 mg/L)	0.4 µg/mg
				100 µg/L SeNPs + 2,4D +BA (0.5 + 3 mg/L)	0.79 µg/mg
Caffeic acid	C_9_H_8_O_4_	180.16	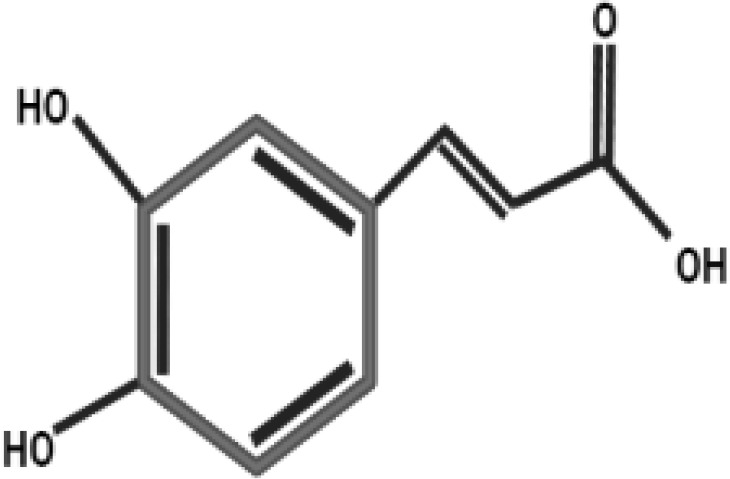	2,4D + BA (0.5 + 3 mg/L)	0.27 µg/mg
				100 µg/L SeNPs + 2,4D + BA (0.5 + 3 mg/L)	0.092 µg/mg
Ferulic acid	C_10_H_10_O_4_	194.18	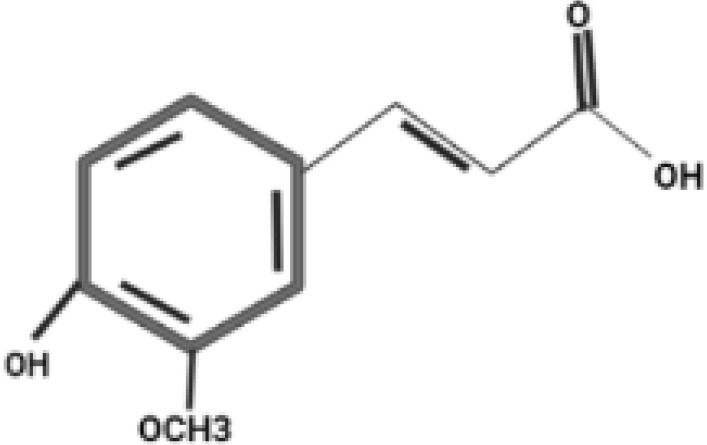	2,4D + BA (0.5 + 3 mg/L)	1.95 µg/mg
				100 µg/L SeNPs + 2,4D + BA (0.5 + 3 mg/L)	2.43 µg/mg
Catechin	C_15_H_14_O_6_	290.27	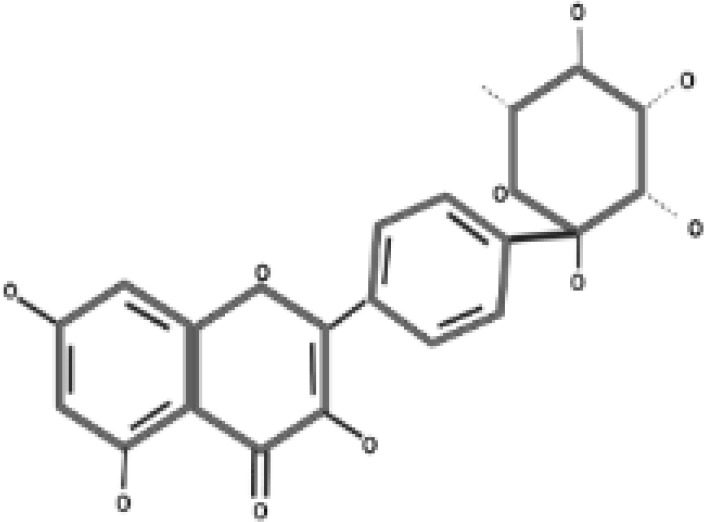	2,4D +BA (0.5 + 3 mg/L)	0.38 µg/mg
				100 µg/L SeNPs + 2,4D + BA (0.5 + 3 mg/L)	0.79 µg/mg
Querctin	C_15_H_10_O_7_	302.23	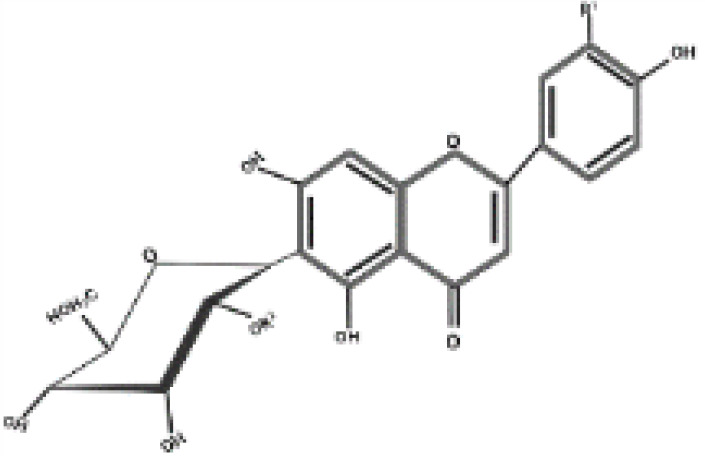	2,4D + BA (0.5 + 3 mg/L)	0.77 µg/mg
				100 µg/L SeNPs + 2,4D + BA (0.5 + 3 mg/L)	1.22 µg/mg
Rutin	C_27_H_30_O_16_	610.5	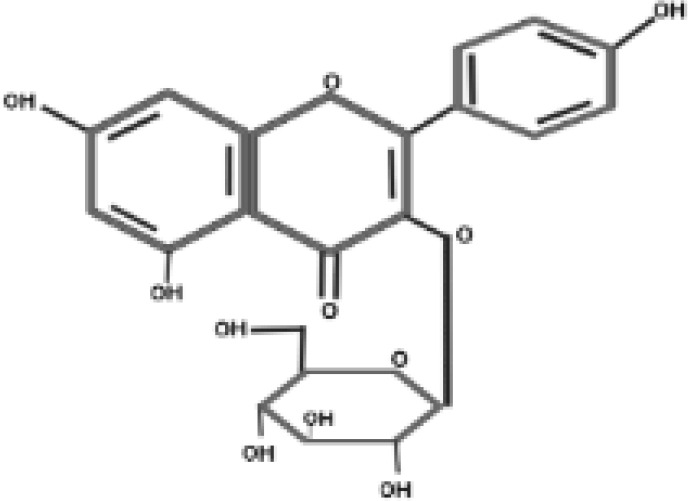	2,4D + BA (0.5 + 3 mg/L)	0.58 µg/mg
				100 µg/L SeNPs + 2,4D + BA (0.5 + 3 mg/L)	0.93 µg/mg

## Conclusions

Given that it is a highly threatened plant in Pakistan, the main goal of this research was to launch a protocol that could permit for the conservation of this rare plant, perpetual callus biomass, and bioactive compound production through *in vitro* callus cultures of *C. tuberculata*. The findings from this project established a first comprehensive assessment regarding the interactive influence of SeNPs along with light regimes on the *in vitro* callus culture’s growth and their antioxidant activities. Calli cultured on media containing SeNPs at 100 µg/L along with PGRs and grown for 2 weeks under a completely dark environment, followed by normal light, were found to be worthwhile sources for callus proliferation and secondary metabolite production. Moreover, the collaborative impact of SeNPs and light regimes proved to be an effective strategy for enhancing the activities of enzymatic antioxidants in *in vitro* callus cultures. LC/ESI-MS/MS data also offer evidence demonstrating that the elicitation of *C. tuberculata* callus with SeNPs at 100 µg/L along with PGRs and for a period of complete darkness for 2 weeks and then exposure to normal light is a viable, safe, and sustainable way of producing bioactive antidiabetic compounds. To discourse several health-related and commercial issues, this elicitation technology is considered the effective mode for the improved production of bioactive metabolites. In the future, the continuation of this study is necessary to explicate the combinatorial influence of SeNPs and light regimes on the production of phytochemical bioactive metabolites at the molecular level.

## Data availability statement

The data presented in the study are deposited in the ProteomeXChange consortium (iProX) repository, accession number IPX0007104000 (https://www.iprox.cn/page/project.html?id=IPX0007104000).

## Author contributions

AAl and Z-u-RM designed the research. AA and SM collected the data. AAl, Z-u-RM, and SM processed the data. AA and JL-A draw and beautify the data images. AAl wrote the original manuscript. JL-A, NR, AAl, and PK provided suggestions and wrote, reviewed, edited, validated, and improved the manuscript. Z-u-RM provided resources and supervision. AAh validated, wrote, and reviewed the manuscript. All authors contributed to the article and approved the submitted version.
