# Supplementary material for: Phytomediated selenium nanoparticles and light regimes elicited in vitro callus cultures for biomass accumulation and secondary metabolite production in Caralluma tuberculata
Source: Front Plant Sci. 2023 Sep 22;14:1253193. doi: 10.3389/fpls.2023.1253193 (PMC10556749; doi:10.3389/fpls.2023.1253193)
Supplement: Supplementary file 1 [file Image_1.pdf]

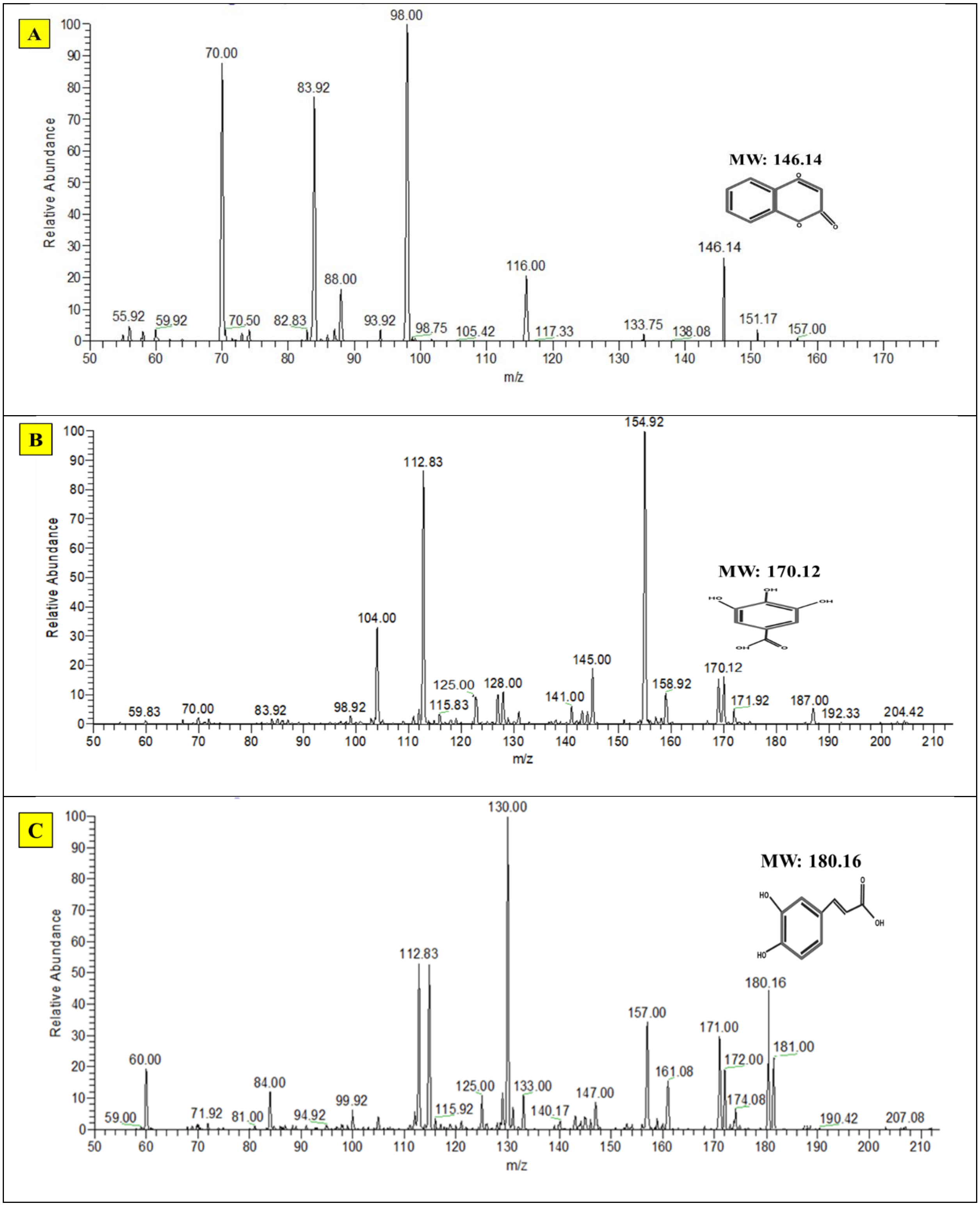
A
MW: 146.14
Relative Abundance
m/z
70.00
83.92
98.00
88.00
116.00
146.14
55.92
59.92
70.50
82.83
93.92
98.75
105.42
117.33
133.75
138.08
151.17
157.00
B
MW: 170.12
Relative Abundance
m/z
154.92
112.83
104.00
125.00
115.83
128.00
141.00
145.00
158.92
170.12
171.92
187.00
192.33
204.42
59.83
70.00
83.92
98.92
C
MW: 180.16
Relative Abundance
m/z
130.00
112.83
180.16
157.00
171.00
172.00
181.00
161.08
174.08
60.00
84.00
99.92
125.00
133.00
147.00
115.92
140.17
59.00
71.92
81.00
94.92
190.42
207.08

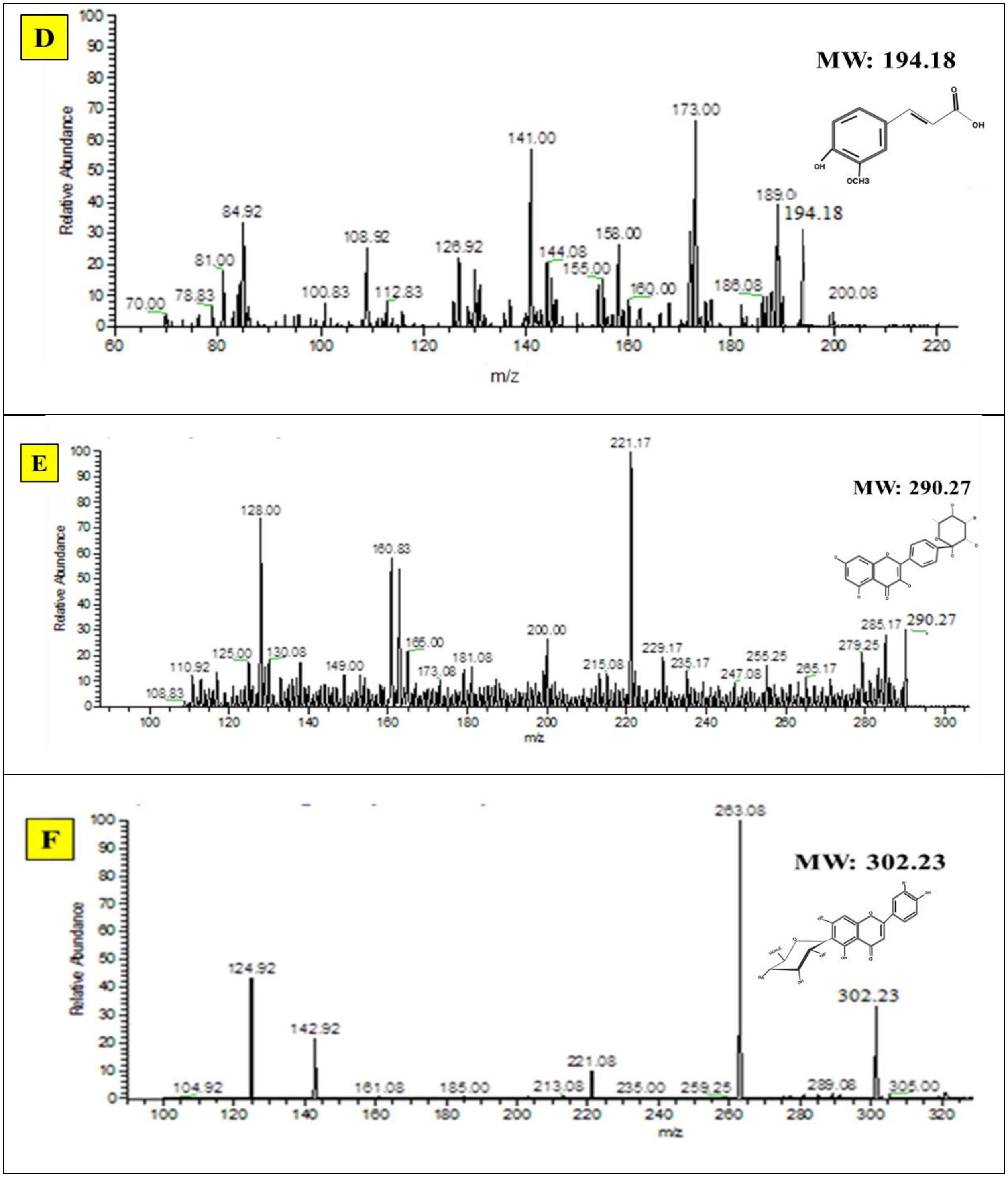
D
MW: 194.18
Relative Abundance
m/z
OH
OCH3
70.00
78.83
81.00
84.92
100.83
108.92
112.83
126.92
141.00
144.08
155.00
158.00
160.00
173.00
186.08
189.0
194.18
200.08
E
MW: 290.27
Relative Abundance
m/z
108.83
110.92
125.00
128.00
130.08
149.00
160.83
165.00
173.08
181.08
200.00
215.08
221.17
229.17
235.17
247.08
255.25
265.17
279.25
285.17
290.27
F
MW: 302.23
Relative Abundance
m/z
104.92
124.92
142.92
161.08
185.00
213.08
221.08
235.00
259.25
263.08
289.08
302.23
305.00

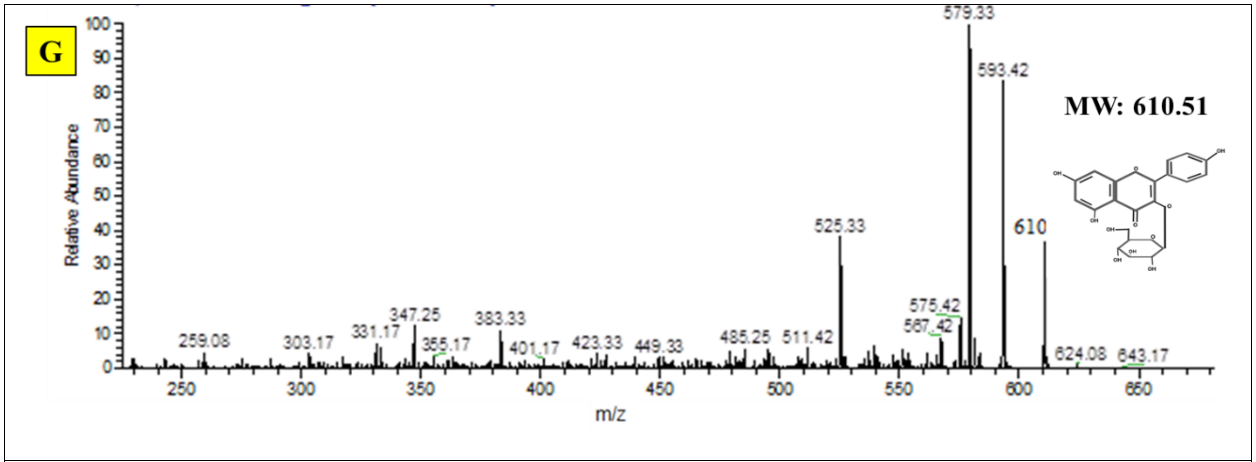

Supplementary Figure 1. Mass spectrum of bioactive antidiabetic compounds in *in vitro* raised *Caralluma tuberculata* callus methanolic extracts scrutinized by liquid chromatography-electrospray ionization-tandem mass spectrometry (LC-ESI-MS/MS). Label: A: Coumarins, B: Gallic acid C: caffeic acid, D: Ferulic acid, E: Catechin F: Querctin, G: Rutin
